# Leveraging biomarkers and primary care embedding for scalable precision cancer prevention in China: Insights from the FuSion study

**DOI:** 10.1016/j.xinn.2025.101189

**Published:** 2025-11-25

**Authors:** Huangbo Yuan, Yanfeng Jiang, Zhenqiu Liu, Renjia Zhao, Ziyu Yuan, Qiuhong Man, Shaohui Liu, Hongxuan Zhang, Lijuan Xu, Yunzhi Zhang, Zhixi Su, Qiye He, Kelin Xu, Tiejun Zhang, Li Jin, Ming Lu, Weimin Ye, Jiangli Zhang, Rui Liu, Chen Suo, Xingdong Chen

**Affiliations:** 1 State Key Laboratory of Genetics and Development of Complex Phenotypes, Human Phenome Institute, Research and Innovation Center, Shanghai Pudong Hospital, Zhangjiang Fudan International Innovation Center, and National Clinical Research Center for Aging and Medicine, Fudan University, Shanghai 200433, China; 2 Fudan University Taizhou Institute of Health Sciences, Taizhou 225300, China; 3 Department of Laboratory Medicine, Shanghai Fourth People’s Hospital, School of Medicine, Tongji University, Shanghai 200434, China; 4 Health Management Center, Xiangya Hospital, Central South University, Changsha 410083, China; 5 Guangdong Cadre Health Management Center, Guangdong Provincial People’s Hospital, Guangdong Academy of Medical Sciences, Guangzhou 510080, China; 6 Health Management Center, Renmin Hospital of Wuhan University, Wuhan 430060, China; 7 Singlera Genomics (Shanghai), Ltd., Shanghai 200120, China; 8 Ministry of Education Key Laboratory of Public Health Safety, School of Public Health, Fudan University, Shanghai 200032, China; 9 Clinical Epidemiology Unit, Qilu Hospital of Shandong University, Jinan 250012, China; 10 Department of Medical Epidemiology and Biostatistics, Karolinska Institute, 17177 Stockholm, Sweden

## From population to individual cancer prevention

Cancer is a leading cause of death in China, with an estimated 2.57 million deaths in 2022.^1^ Despite national screening efforts, most strategies remain age-based or rely on non-specific indicators such as lifestyle or regional incidence, offering limited biological specificity and insufficiently accounting for population heterogeneity. Precision prevention integrates clinical, behavioral, molecular, and environmental data to enable biologically informed risk stratification and targeted interventions,^2^ but scalable deployment requires platforms that link discovery, validation, and primary-care implementation. Despite progress from UK Biobank and Biobank Japan, few systems—globally or in China—combine long-term follow-up, deep molecular profiling, and clinical integration for prevention at scale.

**Figure 1:**
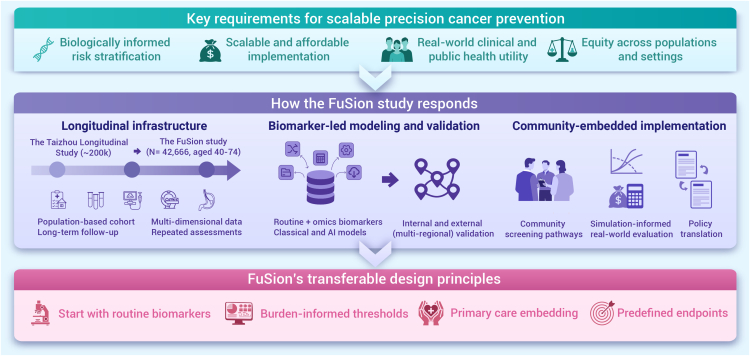
The FuSion framework for scalable precision cancer prevention

To address these gaps and support the practical deployment of precision cancer prevention in China, we initiated the FuSion (Fudan-Singlera for Cancer Early Detection) study in 2021 in Taizhou, Jiangsu Province. Building upon the Taizhou Longitudinal Study (TZL), FuSion has enrolled 42,666 adults aged 40–74 with more than a decade of follow-up. It integrates deep phenotyping, clinical screening, and multi-level analytics to support a three-stage implementation framework: (1) biomarker discovery and validation, (2) risk model deployment in community screening, and (3) translation into public health interventions. FuSion serves not only as a research cohort but also as a delivery-oriented platform designed to meet the practical demands of precision, affordability, and equity in cancer prevention (Figure 1).

## Routine biomarkers for scalable risk stratification

Multi-omics and liquid biopsy offer promise for early detection but are costly and largely inaccessible for large-scale use, especially in resource-limited settings.^3^ Routine biomarkers are already embedded in primary care and could enable low-cost population stratification, yet their panel-level predictive utility remains underexplored.

FuSion evaluated 54 routinely measured biomarkers, including hepatic, metabolic, endocrine, inflammatory, and tumor-related markers, across the cohort.^4^ By 2022, over 1,900 newly diagnosed cancer cases enabled robust site-specific signals (e.g., liver enzymes/insulin/hepatitis markers for liver cancer; pepsinogen I/II ratio and squamous cell carcinoma antigen for upper gastrointestinal malignancies), informing a stratification model now under validation.^4^ In the first-round screening, the highest-risk tier showed three- to seventeen-fold higher detection of precancerous lesions and early-stage cancers, underscoring translational potential.^4^

To mitigate false positives, overdiagnosis, and limited actionability, FuSion uses decision-curve analysis to set thresholds under capacity and equity constraints, and microsimulation/budget-impact modeling to compare screening bundles (questionnaires + biomarkers, with selective imaging as confirmatory testing) over a 5-year horizon. This framework informs protocol design and recalibration, while establishing a pragmatic pathway to selectively incorporate multi-omics and liquid biopsy–based multi-cancer early detection (MCED) tools in the future.

## Translational platform from discovery to action

China has made major progress in large-scale cohorts for chronic disease research. Pioneering efforts such as the China Kadoorie Biobank and other regional longitudinal studies have generated insights into lifestyle and environmental determinants of disease.^5^ However, many lack standardized clinical screening, repeated phenotyping, multi-omics profiling, and subclinical disease detection for translational cancer prevention.

FuSion embeds these capabilities within community-based healthcare systems. Participants complete resurveys with standardized screening (e.g., low-dose CT, ultrasound, endoscopy, breast imaging), physical exams, digital questionnaires, and biospecimen collection (blood, urine, saliva, stool). Around 30,000 participants have completed the first-round resurvey with remeasurement of routine biomarkers. Baseline routine biomarkers and genotyping have been completed for all participants. Ongoing multi-omics assays (whole genome sequencing, proteomics, metabolomics, ctDNA methylation) on baseline biospecimens aim to identify cost-effective stratification targets. These data are integrated with health records to support modeling across biological and clinical domains.

Building on this infrastructure, FuSion evaluates real-world utility and long-term benefits of multi-omics and liquid biopsy technologies in cancer prevention. Classical statistical methods and artificial intelligence (AI) models are applied separately and comparatively to assess the added predictive value of questionnaires, clinical variables, routine biomarkers, and multi-omics features. Model selection is based on discrimination, calibration, and decision-curve net benefit, and models are tested across populations with varying health literacy, socioeconomic status, and healthcare access. To ensure clinical relevance, FuSion prioritizes cancers with established or emerging interventions, stratifies risk into clinically meaningful tiers, and selects biomarkers that can guide post-stratification steps such as counseling, education, surveillance, and referral.

## Embedding cancer risk stratification in primary care

Delivering precision cancer prevention requires integration into scalable, equitable, and locally responsive healthcare systems. FuSion embeds enrollment, screening, and follow-up within township and community health centers, aligning workflows with primary care to evaluate the feasibility and effectiveness of stratification tools.

FuSion applies a two-tiered validation strategy to ensure robustness. Models are trained using data from the initial recruitment wave (2011–2014) and prospectively validated in the later wave (2018–2021), where programmatic screening is linked to diagnostic confirmation and pathology verification. External validation leverages independent community and health-exam cohorts across multiple provinces. Outcomes are primarily ascertained through health records and surveillance systems, supplemented by expert adjudication and histopathologic confirmation.

Endpoints are phase specific: model development focused on 5-year invasive-cancer incidence via registry linkage; prospective internal validation on first-round yield of stage I–II cancers and high-grade premalignant lesions, reflecting early, actionable outcomes; and long-term endpoints, including late-stage incidence and cancer-specific mortality, to evaluate delayed outcomes and potential overdiagnosis. External cohorts use similar invasive cancer endpoints, incorporating staging when available.

## Scalable policy blueprint for precision cancer prevention

As precision cancer prevention transitions from concept to practice, the key challenge is to build usable, affordable, and scalable pathways within routine care. FuSion functions as a community-embedded translational platform that connects discovery, validation, and implementation through deep phenotyping, resurveys, and integrated screening. It prospectively evaluates biomarkers and models against predefined clinical and operational endpoints, enabling data-informed stratification aligned with local capacity and equity constraints.

FuSion provides a practical framework for implementing precision prevention into real-world health systems. Core transferable design principles include:

(1) start with routine, widely available biomarkers while selectively adding omics tools as feasible; (2) set thresholds and biomarker panels based on disease burden, feasibility, and equity; (3) embed survey–screen–follow-up workflows through local partnerships; (4) predefine endpoints and analytic plans based on standardized diagnostic confirmation. To address adherence, data quality, and sustainability, FuSion combines community engagement and logistical support (including transportation assistance and flexible scheduling), harmonized protocols with multi-source verification, and durable government–institution partnerships supported by an on-site team and diversified funding.

Distinct from discovery-oriented observational platforms (e.g., UK Biobank, Biobank Japan), FuSion is cancer-focused and delivery-oriented within primary care, enabling dynamic stratification, pathway testing, and real-world outcome evaluation. Although initially localized, limitations in early generalizability and service capacity are being mitigated through multi-provincial validation, protocol harmonization, and adaptive scale-up.

In sum, scientific innovation alone is not sufficient. Without clear intervention pathways, community engagement, and equity safeguards, precision prevention risks becoming inequitably distributed, thereby undermining its potential as a public good. FuSion demonstrates how technical workflows and social infrastructures can be embedded into health systems, offering a scalable, policy-informed model adaptable to diverse national contexts.

## Funding and acknowledgments

This work was supported by the Science and Technology Innovation 2030 Major Projects (2023ZD0510000), the Shanghai Municipal Science and Technology Major Project (2023SHZDZX02), and the 10.13039/501100001809National Natural Science Foundation of China (82473700).

## Declaration of interests

Z.S., Q.H., J.Z., and R.L. are employees of Singlera Genomics (Shanghai), Ltd.

## Declaration of generative AI and AI-assisted technologies in the writing process

During the preparation of this work, the authors used ChatGPT in order to improve language and readability. After using this tool, the authors reviewed and edited the content as needed and take full responsibility for the content of the publication.
